# Корреляция исходов лечения бесплодия методом экстракорпорального оплодотворения (ЭКО) и массы тела женщин репродуктивного возраста.

**DOI:** 10.14341/probl12727

**Published:** 2021-01-25

**Authors:** А. С. Дружинина, И. И. Витязева, Д. А. Димитрова

**Affiliations:** Национальный медицинский исследовательский центр эндокринологии; Национальный медицинский исследовательский центр эндокринологии; Национальный медицинский исследовательский центр эндокринологии

**Keywords:** Избыточная масса тела, ИМТ, ожирение, беременность после ЭКО

## Abstract

**Обоснование:**

Обоснование. Ожирение/избыточная масса тела (ИзбМТ) у женщин часто являются причинами нарушения менструальной функции и бесплодия.

**Цель:**

Цель. Установить корреляцию между ожирением/ИзбМТ и эффективностью лечения бесплодия методом экстракорпорального оплодотворения (ЭКО) по частоте наступления клинической беременности, ее исходам и массе плода при рождении.

**Методы:**

Методы. Ретроспективно проанализированы данные историй болезни 1874 пациенток, которым проведено лечение бесплодия методом ЭКО в период 01.2012–12.2019 гг. Критерии исключения: дефицит массы тела, синдром поликистозных яичников, программы с использованием донорских ооцитов, эктопические беременности, оплодотворение эпидидимальными/тестикулярными сперматозоидами партнера. В исследование включены 1583 пациентки в возрасте 21–45 лет (медиана 33,0 года [30,0; 37,0]). Статистическая обработка данных проводилась с использованием пакета прикладных программ STATISTICA (StatSoft). Пороговый уровень статистической значимости p<0,05.

**Результаты:**

Результаты. До начала индукции суперовуляции в протоколе ЭКО пациенткам проводилось антропометрическое исследование: измерение роста и веса с расчетом индекса массы тела (ИМТ) (медиана — 23 кг/м2 [20,7; 26,2]). Пациентки были разделены на 5 групп в зависимости от показателя ИМТ: группа 1 с нормальной массой тела (НМТ) — n=1061, группа 2 (ИзбМТ) — n=368, группа 3 (ожирение I ст.) — n=117, группа 4 (ожирение II ст.) — n=36, группа 5 (ожирение III ст.) — n=1. В каждой группе оценивались частота наступления клинической беременности (ЧНКБ) и ее исход: частота самопроизвольных абортов (СА), преждевременных (ПР), своевременных родов (СР) маловесными детьми (масса при рождении <2500 г), новорожденных с НМТ (2500–3999 г) и родов крупным плодом (ЧРКП) (≥4000 г) среди пациенток с одноплодной беременностью. ЧНКБ по группам статистически не различалась: 34,6, 34,5, 30,7, 41,7%, у пациентки группы 5 наступила маточная ­одноплодная беременность, исход остался неизвестным. В результате лечения наступило 542 беременности: 407 одноплодных (74,4%), 132 двойни (24,1%) и 3 тройни (0,5%). СР при одноплодной беременности: 71,9, 67,6, 70,8, 60,0%; ПР — 7,7, 5,4, 8,3, 0,0%; СА в I триместре беременности — 18,3, 25,7, 20,8, 40,0%; СА во II триместре — 2,13%, по 1,4% в группах 2, 3 и 4 соответственно. Частота родов маловесными детьми — 8,8, 11,4, 6,3, 0%; новорожденные с НМТ — 84,9, 84,1, 75,0, 60,0%; ЧРКП — 6,3, 4,6, 18,8, 40,0% соответственно.

**Заключение:**

Заключение. При проведении корреляционного анализа зависимости ЧНКБ и ее исходов от ИМТ пациентки не выявлено (р=0,975 и р=0,469 соответственно). ЧРКП достоверно чаще встречалась у пациенток с ожирением (р=0,0016). Необходимо провести дальнейшие исследования, используя новые критерии формирования групп для получения углубленных результатов.

## Abstract (en)

**Backgraund:**

Backgraund: obesity/overweight in women are often the causes of menstrual dysfunction and infertility.

**Aims:**

Aims: To identify the association between overweight/obesity and IVF outcomes.

**Materials and methods:**

Materials and methods: retrospective study — data of 1874 patients undergoing IVF in the Endocrinology Research Centre (2012–2019) was analyzed. Exclusion criteria: BMI <18.5 kg/m2, polycystic ovary syndrome, donation of ­oocytes, ectopic pregnancy, fertilization with partner’s epididymal/testicular sperm. The study included 1583 women aged 21–45 years (median 33.0 y.o. [30.0; 37.0], median BMI 23 kg/m2 [20.7; 26.2]). Statistical data processing was performed using the STATISTICA application package (StatSoft). The threshold level of statistical significance is <0.05.

**Results:**

Results: Patients were divided into 5 groups (gr.): normal body weight (NBW) - 1061 people (ppl.) (gr. 1), overweight — 368 (gr. 2), class I obesity — 117 (gr. 3), class II obesity — 36 (gr. 4), class III obesity — 1 (gr. 5). In each group, the estimated pregnancy rate (PR) and its outcomes, the frequency of lightweight newborns (body weight at birth <2500g), newborns with NBW (2500-3999g), births with a large fetus (≥4000g) were measured. The PR didn’t differ: 34.6%, 34.5%, 30,7%, 41,7%, respectively, the woman in gr.5 got pregnant. Among 407 (74.4%) singleton pregnancies urgent delivery was registered in 71.91%, 67,57%, 70,83%, 60,0%, gr. 5 — no ­information. Premature birth: 7,66%, 5,41%, 8,33%, 0%. Spontaneous abortion in the 1st trimester: 18,30%, 25,68%, 20,83%, 40,0%. Spontaneous abortion in the 2nd trimester: 2,13%, 1,35% in gr. 2, 3, 4. Lightweight newborns: 8,81%, 11,36%, 6,25%, 0%. Newborns with NBW: 84,91%, 84,09%, 75,0%, 60,0%. Large-childbirth — 6,29%, 4,55%, 18,75%, 40,0%.

**Conclusions:**

Conclusions: Correlation analysis of the dependence of PR and its outcomes on the BMI was not revealed (p=0.975 and p=0.469, respectively). Large fetus births were more often detected in obese patients (p=0.0016). A large prospective group is needed to expand the estimated body parameters to the IVF outcomes.

## ОБОСНОВАНИЕ

Проблема ожирения во всем мире за последние десятилетия занимает одно из первых мест по распространенности и не имеет тенденции к снижению [1][2]. По данным ВОЗ (2018), 650 млн (13%) населения страдают ожирением (11% мужчин и 15% женщин), более 1,9 млрд (39%) взрослых старше 18 лет (39% мужчин и 40% женщин) имеют избыточный вес. С 1975 по 2016 гг. число людей, страдающих ожирением, во всем мире выросло более чем втрое [3]. Это касается лиц обоих полов и всех возрастных групп, при этом более половины женщин репродуктивного возраста имеют избыточный вес (индекс массы тела (ИМТ) ≥25 кг/м2) или страдают ожирением (ИМТ ≥30 кг/м2) [4].

Ожирение, в основном из-за хронической ановуляции, приводит к нарушению фертильности женщины [5]. Многими авторами отмечается увеличение продолжительности периода до наступления беременности у женщин с ожирением по сравнению с нормовесными, даже в случае регулярности менструального цикла и наличия овуляции. Ожирение у женщин связано с нарушением менструальной функции, ановуляцией, бесплодием, повышенным риском развития гиперплазии эндометрия, эмбриологическими особенностями и клиническими параметрами стимуляции в протоколах экстракорпорального оплодотворения (ЭКО) [6][7]. Ожирение у женщин напрямую связано с неблагоприятными исходами беременности, такими как самопроизвольное прерывание, внутриутробная задержка развития плода, гипертония, преэклампсия и гестационный сахарный диабет [8][9].

Превышение порогового значения ИМТ является фактором риска развития таких состояний, как заболевания сердечно-сосудистой системы, сахарный диабет (СД) 2 типа (СД2), патология опорно-двигательного аппарата, онкологические заболевания. Развитие данных состояний у взрослого населения репродуктивного возраста ведет к повышению инвалидизации в молодом возрасте и, как следствие, снижению рождаемости [2].

С каждым годом распространенность ожирения среди женщин репродуктивного возраста неуклонно растет. В структуре бесплодного брака частота встречаемости эндокринного фактора бесплодия составляет 25% [10][11].

Здоровый образ жизни, правильное питание, поддержание ИМТ от 19 до 30 кг/м2 увеличивают вероятность зачатия, в то время как период до зачатия у женщин с ИМТ>35 кг/м2 увеличивается в 2 раза [12].

По данным мировой литературы, ожирение оказывает неблагоприятное влияние на исходы как спонтанных беременностей, так и беременностей, наступивших в результате ЭКО. Однако гетерогенность исследуемых групп пациенток и оцениваемых параметров не позволяет сделать однозначные выводы. Данные о влиянии ожирения на показатель рождаемости также противоречивы. Например, по данным метаанализа Rittenberg V. и соавт. (2011), у женщин с избыточной массой тела (ИзбМТ) или ожирением (ИМТ ≥25 кг/м2) частота наступления клинической беременности (ЧНКБ) (относительный риск (ОР)=0,90; р<0,0001) и живорождения была значительно ниже (ОР=0,84; р=0,0002), а также значительно выше частота самопроизвольных абортов (СА) (ОР=1,31); р<0,0001) по сравнению с женщинами с нормальной массой тела (НМТ) [13]. По результатам исследования MacKenna A. и соавт. (2017) было обнаружено, что показатель ИМТ не был связан с частотой наступления беременности, живорождения и преждевременного прерывания беременности [14].

По мнению отечественных и зарубежных специалистов, частота бесплодия у женщин с ожирением составляет 33,6% по сравнению с 18,6% женщин с НМТ [7].

Частота развития ожирения у детей, рожденных от матерей с ИМТ>30 кг/м2, выше, чем у пациенток с НМТ [3]. По данным шведских ученых, у детей, рожденных от родителей с ожирением, отмечается ухудшение показателей метаболизма: возрастает частота ожирения, СД, нарушений репродуктивной функции, а также аутизма [15].

Учитывая длительность терапии ожирения, зачастую низкую приверженность пациенток к лечению, достаточно сложно добиться снижения массы тела перед вступлением в протокол ЭКО. Стоит отметить также группу пациенток старшего репродуктивного возраста со сниженным овариальным резервом, когда длительное снижение массы тела может означать путь к использованию донорских ооцитов.

## ЦЕЛЬ

Выявить корреляцию между ожирением/избыточной массой тела и результатами лечения бесплодия методом ЭКО по ЧНКБ, ее исходам и весу новорожденных детей.

## МЕТОДЫ

## Дизайн исследования

В ретроспективное интервенционное (экспериментальное) одноцентровое выборочное неослепленное рандомизированное исследование были включены 1583 пациентки с бесплодием, обратившиеся для лечения методом ЭКО в отделение ВРТ ФГБУ «НМИЦ эндокринологии» Минздрава России. Медиана возраста составила 33,0 года [ 30,0; 37,0 ].

## Критерии соответствия

Критерии исключения: дефицит массы тела (ИМТ<18,5 кг/м2), наличие синдрома поликистозных яичников, программы с использованием донорских ооцитов, эктопические беременности, оплодотворение эпидидимальными/тестикулярными сперматозоидами.

## Условия проведения

В исследование включались только пациентки, подвергшиеся лечению методом ЭКО в ФГБУ «НМИЦ эндокринологии» Минздрава России.

## Продолжительность исследования

Период включения составил 96 мес (8 лет), c 2012 по 2019 гг.

## Описание медицинского вмешательства

В день вступления в протокол ЭКО (на 2-й день менструального цикла) после предварительного обследования, согласно Приказу №107н МЗ РФ от 12 февраля 2013 г., сбора анамнеза, общего и гинекологического осмотров, для выявления противопоказаний к лечению — ультразвукового исследования органов малого таза, получения информированного добровольного согласия пациентки и ее мужа (партнера) на проведение лечения бесплодного брака методом ЭКО/ЭКО-ИКСИ пациенткам проводилось антропометрическое исследование: измерение роста и веса с последующим расчетом ИМТ (медиана ИМТ — 23 кг/м2 [ 20,7; 26,2 ]).

## Основной исход исследования

В ходе исследования проводилась оценка ЧНКБ, ее исходов и родов маловесными и крупными плодами среди одноплодных беременностей.

## Дополнительные исходы исследования

Дополнительных исходов исследования не отмечалось.

## Анализ в подгруппах

Пациентки были разделены на 5 групп в зависимости от ИМТ: группа (гр.) 1 — с НМТ — 1061 человек (67,00%), гр. 2 — ИзбМТ — 368 (23,24%), гр. 3 — ожирение I ст. — 117 (7,39%), гр. 4 — ожирение II ст. — 36 (2,27%), гр. 5 — ожирение III ст. — 1 (0,06%). Распределение пациенток по ИМТ представлено на рисунке 1.

**Figure:**
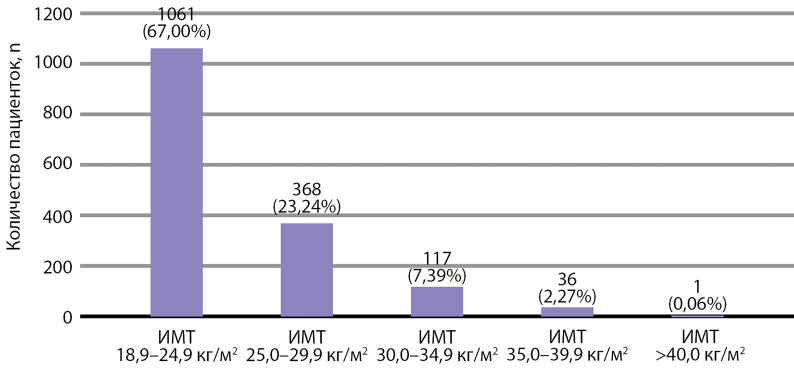
Рисунок 1. Количество пациенток (n) в зависимости от индекса массы тела.

В каждой группе оценивалась ЧНКБ, ее исходы, а также частота рождения маловесных детей (масса тела при рождении <2500 г), новорожденных с НМТ (2500–3999 г), частота родов крупным плодом (ЧРКП; ≥4000 г) среди пациенток с одноплодной беременностью.

## Методы регистрации исходов

С целью регистрации факта наступления беременности использовались данные анализа сыворотки крови на хорионический гонадотропин человека (ХГч). Подтверждение факта наступления маточной беременности проводилось методом УЗИ органов малого таза на 19–21-й день после переноса эмбриона/-ов, факт исходов беременности — с помощью средств коммуникации (телефон, электронная почта).

## Этическая экспертиза

Локальный этический комитет при ФГБУ «НМИЦ эндокринологии» Минздрава России (выписка из протокола №12 от 27.06.2018 г.) постановил одобрить возможность проведения научно-исследовательской работы по теме «Разработка методов ведения пациентов с эндокринопатиями и бесплодием с учетом постнатальных исходов в программах вспомогательных репродуктивных технологий».

## Статистический анализ

Принципы расчета размера выборки. Размер выборки предварительно не рассчитывался.

Методы статистического анализа данных. Статистический анализ данных выполнялся с использованием пакета программ Statistica 9.1 (StatSoft, Inc., США) в соответствии с рекомендациями. Описательная статистика количественных признаков представлена средними и среднеквадратическими отклонениями (в формате M±SD; в случае нормальных распределений) либо медианами и квартилями (в формате Me [Q1; Q3]). Описательная статистика качественных признаков представлена абсолютными и относительными частотами. Для оценки ассоциации бинарных признаков использовался двусторонний точный критерий Фишера (two-tailed Fisher exact test). Пороговым уровнем статистической значимости считали 0,05.

## РЕЗУЛЬТАТЫ

## Объекты (участники) исследования

Объектами исследования являются источники данных — медицинские карты пациенток.

## Основные результаты исследования

ЧНКБ в зависимости от уровня ИМТ статистически не различалась: в гр. 1 этот показатель составил 34,6%, в гр. 2 — 34,5%, в гр. 3 — 30,7%, в гр. 4 — 41,7% соответственно, у женщины с ожирением III степени также была подтверждена маточная беременность. ЧНКБ по группам в зависимости от ИМТ представлена на рисунке 2.

Установлен факт 407 родов одним плодом (74,4%), 132 — двойней (24,1%) и 3 — тройней (0,5%). Среди одноплодных беременностей своевременные роды (СР) были зарегистрированы в гр. 1 у 169 женщин (71,91%), в гр. 2 — 50 (67,57%), в гр. 3 — 17 (70,83%), в гр. 4 — 6 (60,0%) соответственно. Не было получено информации об исходе беременности у пациентки в группе 5. Преждевременные роды (ПР) зарегистрированы в гр. 1 у 18 женщин (7,66%), в гр. 2 — 4 (5,41%), в гр. 3 — 2 (8,33%), в гр. 4 — 0 (0,0%) соответственно. СА в I триместре беременности выявлены в гр. 1 у 43 женщин (18,30%), в гр. 2 — 19 (25,68%), в гр. 3 — 5 (20,83%), в гр.  4 — 4 (40,0%) соответственно.

СА во II триместре беременности выявлены у 5 человек (2,13%) в гр. 1, по 1 (1,35%) в гр. 2, 3 и 4 соответственно. Исходы клинических беременностей в зависимости от ИМТ матери представлены на рисунке 3.

Роды маловесными детьми среди пациенток с одноплодной беременностью были зарегистрированы у 14  женщин в гр. 1 (8,81%), гр. 2 — 5 (11,36%), гр. 3 — 1 (6,25%), гр. 4 — 0; новорожденных с НМТ в гр. 1 — 135 детей (84,91%), гр. 2 — 37 (84,09%), гр. 3 — 12 (75,0%), гр. 4 — 3 (60,0%); ЧРКП  в гр. 1 — 10 (6,29%), в гр. 2 — 2 (4,55%), в гр.  3 — 32 (18,75%), в гр.  4 — 2 (40,0%) соответственно.

Показатели массы тела новорожденных в зависимости от ИМТ матери представлены на рисунке 4.

**Figure:**
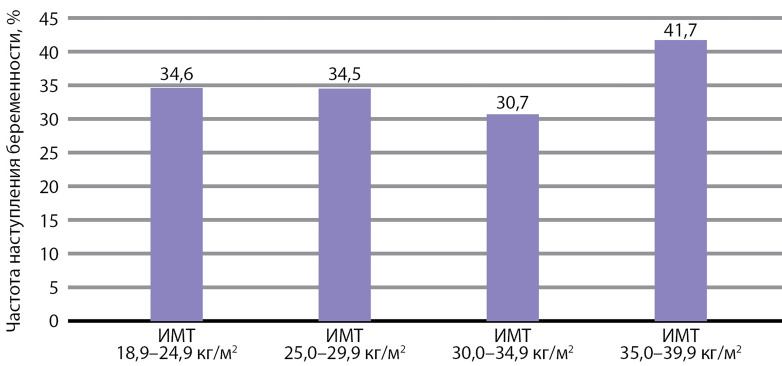
Рисунок 2. Частота наступления клинической беременности в зависимости от индекса массы тела матери (%).

**Figure:**
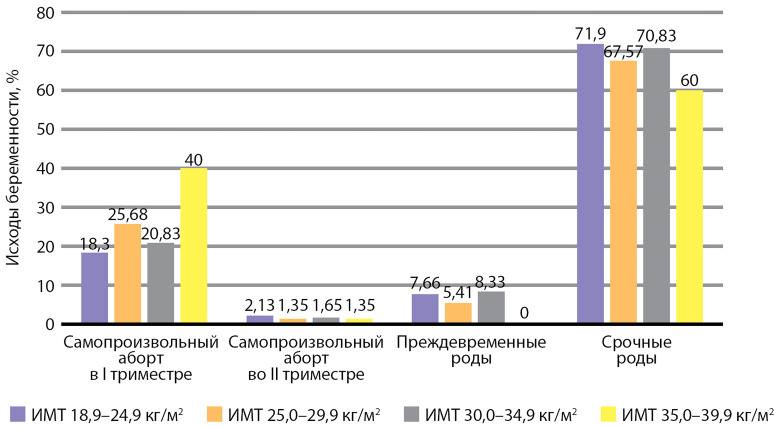
Рисунок 3. Исходы беременности в зависимости от индекса массы тела матери (%).

**Figure:**
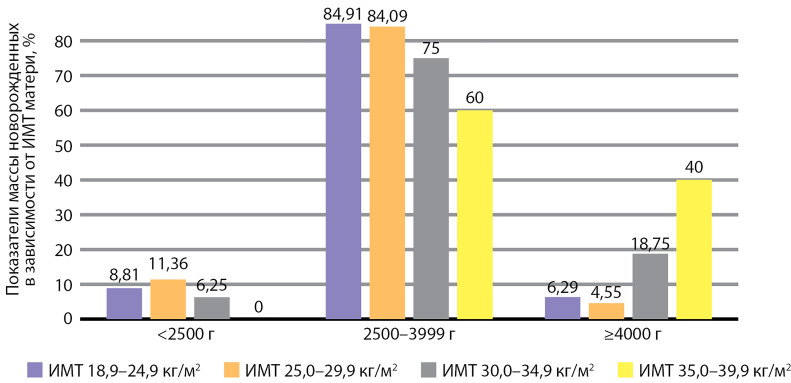
Рисунок 4. Показатели массы новорожденных в зависимости от индекса массы тела матери (%).

## Дополнительные результаты исследования

Дополнительных результатов исследования выявлено не было.

## Нежелательные явления

Нежелательные явления не отмечались.

## ОБСУЖДЕНИЕ

## Обсуждение основного результата исследования

По результатам проведенного исследования ЧНКБ в группах статистически не различалась (р=0,975), однако, возможно, полученные данные связаны с различной численностью выборок групп. Данные зарубежной литературы за 2019 г., посвященные проблеме влияния ИМТ на исходы лечения бесплодия методом ЭКО, противоречивы, но в последнее время появляется все больше сообщений о негативном влиянии ожирения: ухудшается восприимчивость яичников к стимуляции препаратами гонадотропинов, требуя более высоких доз лекарственных препаратов, повышается риск отмены программ лечения в связи со снижением или полным отсутствием овариального ответа, достаточным ростом фолликулов, снижается качество ооцитов и эмбрионов, частота имплантации, ЧНКБ, повышается частота остановки развития на ранних этапах эмбриогенеза, преждевременных родов, низкого веса детей при рождении по сравнению с женщинами с НМТ [7][16][17].

В ходе проведения исследования в отделении ВРТ ФГБУ «НМИЦ эндокринологии» Минздрава России статистической зависимости наступления срочных родов от ИМТ выявлено не было (р=0,469), однако отмечалась тенденция к снижению данного показателя у пациенток с ожирением II степени. При этом частота СА в I триместре беременности имела резкую тенденцию к увеличению также у пациенток с ожирением II степени, однако частота потерь во II триместре беременности преобладала у пациенток с НМТ, что, наиболее вероятно, связано с большим количеством наступивших беременностей в этой группе.

По данным литературы отмечается, что частота имплантации эмбрионов и частота СА напрямую зависят от ИМТ [18]. Также зависимость результатов лечения бесплодия методом ЭКО от ИМТ выявлена в исследовании Zhang и соавт. (2019): снижение частоты имплантации (скорректированное отношение шансов (СОШ) 0,80; 95% ДИ 0,73–0,87), ЧНКБ (СОШ 0,81; 95% ДИ 0,71–0,91) и коэффициента живорождения (СОШ 0,70; 95% ДИ 0,62–0,80). Более того, показатель невынашивания беременности как в I (СОШ 1,46; 95% ДИ 1,15–1,87), так и во II триместрах (ОР 2,76; 95% ДИ 1,67–4,58) был значительно выше у пациенток с ожирением. Но стоит отметить, что авторы использовали классификацию ожирения для азиатской расы, которая имеет незначительные отличия от классификации для европеоидов [19].

Схожие с полученными нами результаты отмечались у китайской группы исследователей: частота рождения живым плодом снижалась у женщин пропорционально увеличению ИМТ [20].

Обращает на себя внимание преобладание рождения детей с массой тела ≥4000 г у пациенток с ожирением I и II степени, что также было показано в зарубежных исследованиях последних 10 лет [21–23]. При изучении влияния ожирения матери до беременности была отмечена связь повышенного риска развития ожирения и ИзбМТ у детей в раннем возрасте, зачатых с помощью ЭКО/ЭКО-ИКСИ, с риском снижения интеллектуальных способностей ребенка [24].

## ЗАКЛЮЧЕНИЕ

В ходе проведения статистического анализа зависимость ЧНКБ и исходов беременности от ИМТ спорна (р=0,486 и 0,469 соответственно). Роды крупным плодом достоверно чаще встречались у пациенток с ожирением (р=0,0016), но не имели достоверного различия от степени ожирения (р=0,159). Следует отметить, что только у 1 пациентки с ожирением III степени результатов исхода беременности получено не было. Таким образом, сказать о влиянии морбидного ожирения на результаты ЭКО не представлялось возможным. Необходимо провести дальнейшие исследования, используя новые критерии формирования групп и введение дополнительных критериев включения/исключения пациенток для получения более углубленных результатов. Также необходимо оценивать степень влияния на исходы ЭКО и беременности диетологической стратегии на этапе предгравидарной подготовки и во время беременности.
